# Adenotonsillectomy and adenoidectomy in children: The impact of timing of surgery and post‐operative outcomes

**DOI:** 10.1111/jpc.16052

**Published:** 2022-06-03

**Authors:** Francisco J Schneuer, Katy JL Bell, Chris Dalton, Adam Elshaug, Natasha Nassar

**Affiliations:** ^1^ The Children's Hospital at Westmead Clinical School Faculty of Medicine and Health, The University of Sydney Sydney New South Wales Australia; ^2^ Sydney School of Public Health Faculty of Medicine and Health, The University of Sydney Sydney New South Wales Australia; ^3^ Bupa ANZ Sydney New South Wales Australia; ^4^ Centre for Health Policy Melbourne School of Population and Global Health, University of Melbourne Melbourne Victoria Australia; ^5^ Menzies Centre for Health Policy and Economics, Charles Perkins Centre Sydney School of Public Health, Faculty of Medicine and Health, The University of Sydney Sydney New South Wales Australia

**Keywords:** adenoidectomy, adenotonsillectomy, Australia, population health, post‐operative, surgery

## Abstract

**Aim:**

To investigate the impact of adenotonsillectomy (ADT) and adenoidectomy (AD) on child health and evaluated their post‐operative complications.

**Methods:**

We included all children aged <16 years undergoing ADT (tonsillectomy ± adenoidectomy) or AD in New South Wales, Australia, 2008–2017. Health information was obtained from administrative hospitalisation data. Rates of post‐operative complications and reoperation were evaluated using generalised estimating equations and Kaplan–Meier methods, respectively.

**Results:**

Out of 156 500 included children, 112 361 had ADT and 44 139 had AD. Population rates increased during 2008–2017 (ADT: 68–79 per 10 000 children; AD: 25–34 per 10 000), and children were increasingly operated on at a younger age. Overall, 7262 (6.5%) and 1276 (2.9%) children had post‐operative complications (mostly haemorrhage), and 4320 (3.8%) and 5394 (12.2%) required reoperation, following ADT and AD, respectively. Complication rates were highest among children aged 0–1 years, lowest for those 2–5 years and increased with age thereafter. Three‐year reoperation rates for children aged 0–1 years were 9.0% and 25.9% following ADT and AD, respectively, decreasing thereafter to 0.5% and 2.1% in children aged 12–13 years.

**Conclusions:**

ADT and AD in Australian children have both increased in frequency and are being done at a younger age. Post‐operative complications and reoperation rates highlight surgery is not without risk, especially for children under 2 years old. These findings support a more conservative approach to management of upper respiratory symptoms, with surgery reserved for cases where potential benefits are most likely to outweigh harms.

## What is already known on this topic

1. Adenotonsillectomy (ADT) and adenoidectomy (AD) are one of the most common operations performed in children.
2. ADT and AD are recommended for recurrent tonsillitis, sleep‐disordered breathing and otitis media, and are not exempt of complications and the need for reoperation.

## What this study adds

1. Population rates of adenotonsillectomy (ADT) and adenoidectomy (AD) in children are rising with children operated increasingly at a younger age.
2. Post‐operative complications were proportionally low but increased with age.
3. One in five children having AD under 5 years required reoperation.
4. Findings highlight that ADT and AD are not without risk, especially for children under 2 years and support a more conservative management approach.

Surgical removal of tonsils (tonsillectomy), adenoids (adenoidectomy (AD)) or both (adenotonsillectomy (ADT)) are the most common operations performed in children, representing a major impact on child health, families and paediatric health care.^1^

Clinical guidelines recommend that tonsillectomy and/or ADT be considered for children presenting with obstructive sleep‐disordered breathing affecting growth, school performance or behaviour, or recurrent throat infections. Obstructive sleep‐disordered breathing is a spectrum of breathing disorders from snoring to the most severe obstructive sleep apnoea (OSA). OSA is characterised by disruption of normal ventilation and sleeping patterns detected with overnight polysomnography.^2^ Parental and clinician concerns about potential long term adverse cardiovascular, cognitive, behavioural and developmental consequences from OSA in children may be driving the increasing use of ADT as the primary line of treatment.^3^ AD may be used for OSA alone if the tonsils are not enlarged, or in the treatment of nasal obstruction, rhinosinusitis and is also recommended as an adjuvant procedure to myringotomy in children with otitis media with effusion and clear signs of adenoid hypertrophy.^4^

However, results regarding the effectiveness of ADT or AD in improving short‐ and long‐term outcomes are mixed. A review of clinical trials suggests that the reduction in throat infections after ADT in the first post‐operative year is moderate, and the benefits do not appear to persist over time.^5^ This overall review finding may be due to differences between the individual studies definition of indications for ADT applied in trials and clinical guidelines, with the greatest benefit found among those children who have more severe and frequent episodes.^6^ For children with mild to moderate OSA aged between 3 and 9 years, clinical trials have reported that ADT led to improvements in quality of life, sleep quality, behaviour and executive function with no improvement in cognition.^7^, ^8^

Potential benefits of removing tonsils and/or adenoids have to be balanced against the risk of post‐operative complications, reoperation, potential long‐term adverse effects,^9^ and the probability that symptoms will naturally resolve. ADT has one of the highest rates of post‐operative readmissions and emergency department visits among paediatric surgical procedures,^1^ and reoperations due to regrowth of tissue or recurrence of symptoms are not uncommon.^10^ In addition, the recent Australian Atlas of Healthcare Variation highlighted potential overuse of ADT and urgent need of contemporary information on short‐ and long‐term outcomes following paediatric ADT.^11^ The aim of the study was to determine the impact of ADT and AD on child health and evaluate post‐operative outcomes.

## Methods

We included all children aged younger than 16 years admitted to hospital to undergo ADT or AD in New South Wales (NSW), Australia between January 2008 and December 2017, and followed them up until June 2018. Health information was ascertained via record linkage of the NSW Admitted Patient Data Collection (APDC). The APDC is a statutory collection of all admissions to public and private hospitals comprising patient demographic information and related diagnoses and procedures coded according to the 10th revision of the International Classification of Diseases, Australian Modification (ICD‐10‐AM) and the Australian Classification of Health Interventions (ACHI), respectively.

ACHI codes for ADT (41789‐01, 41789‐00) and AD (41801‐00) were used to identify the study cohorts. We identified the indication for surgery using the diagnosis fields and categorised them into: (i) chronic diseases of tonsils and adenoids (ICD‐10‐AM: J03 and J35); (ii) sleep‐disordered breathing (G47, R06.5); (iii) ear conditions (H61‐H68) other respiratory conditions (J00, J04, J04, J05, J06 and J37); (iv) peritonsillar abscess (J36); and v) other conditions. Study outcomes included post‐operative complications recorded during the initial admission or during readmission within 30 days following discharge from the index surgery and reoperation in any diagnosis field. Post‐operative complications were defined as haemorrhage, respiratory diseases (e.g. pneumonia and bronchitis), infections or related symptoms (e.g. pain, nausea or vomiting) and adverse effect of anaesthesia or therapeutic drugs. Post‐operative haemorrhage requiring operative procedure was also differentiated. Reoperations were also defined as a subsequent admission for ADT or AD.

Explanatory variables included the calendar year, age, sex, socioeconomic disadvantage, area of residence, hospital type, adjuvant operations and diagnoses of chronic conditions. Socioeconomic disadvantage was determined by recorded postcode of residence using the Socioeconomic Indexes for Areas developed by the Australian Bureau of Statistics (ABS) and classified into quintiles. Adjuvant operations included those involving the nose or sinus (e.g. turbinectomy or septoplasty) and ear operations (e.g. myringotomy). Chronic conditions potentially influencing outcomes were based on diagnoses of neurologic, cardiovascular, respiratory, renal, gastrointestinal, hematologic, metabolic conditions, congenital anomalies and malignancy recorded during the index operation admission or in previous hospital admissions.

### Ethics approval

Ethics approval was obtained from the NSW Population and Health Services Research Ethics Committee (2019/ETH11532).

### Statistical analyses

We evaluated the population rates of ADT and AD by year and demographic characteristics using population data reported by the ABS. Health characteristics of children were evaluated using contingency tables. We calculated the crude rates of post‐operative haemorrhage and any complications by the type of operation, age group and whether children had adjuvant operations. Generalised estimating equations with a logit link and exchangeable correlation were used to standardised rates with 95% confidence intervals (CIs) of post‐operative haemorrhage and any post‐operative complications following ADT and AD, while taking into account explanatory variables and clustering of individuals within hospitals. We determined 1‐, 2‐ and 3‐year reoperation rates with 95% CIs using Kaplan–Meier methods and used multivariable Cox regression to calculate standardised reoperation rates. All analyses were conducted using SAS 9.4 (SAS Institute, Cary, NC, USA).

## Results

**Fig. 1:**
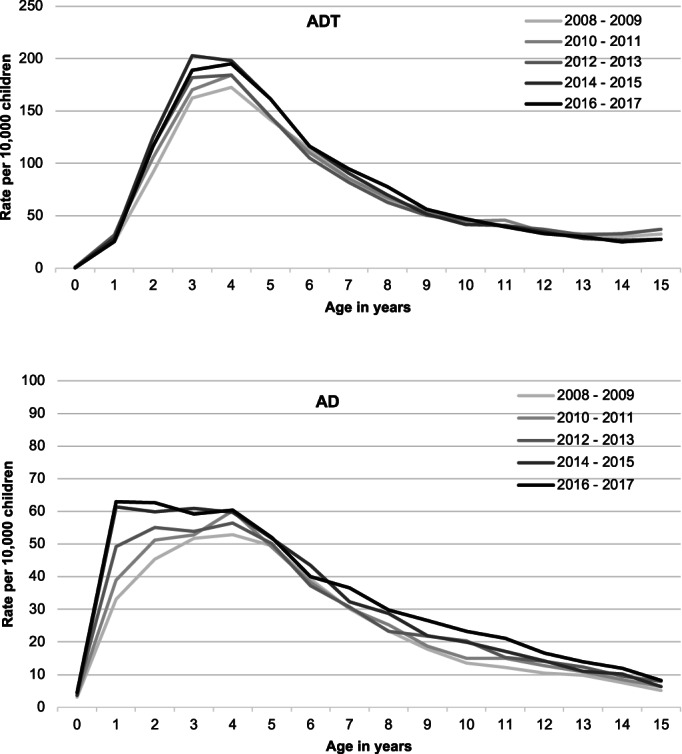
Population rates of operations by age and year of birth in children in NSW, Australia, 2008–2017.

A total of 156 500 children had surgery including 112 361 undergoing ADT and 44 139 AD. Population prevalence of ADT increased from 69 per 10 000 children aged 0–15 years in 2008–2009 to 79 per 10 000 in 2016–2017 and AD increased from 25 to 34 per 10 000 children. There was an overall shift in the age‐specific rates of ADT and AD over time, with children increasingly operated at younger ages in more recent years (Fig. 1). Specifically, children aged 3 years had the largest increase in population rates of ADT (from 162 to 188 per 10 000 children), while there was twofold increase in rates of AD in children aged 1 year (from 33 to 63 per 10 000 children). Population rates were also higher in males and those residing in major cities, lower among children from most disadvantaged backgrounds and nearly two‐thirds of operations were performed in private hospitals (Table 1).

**Table 1:** Demographic and health characteristics of children undergoing tonsils and/or adenoid removal in NSW, 2008–2017

	Adenotonsillectomy, *N* = 112 361	Adenoidectomy, *N* = 44 139
Year	*n* (rate per 10 000 children)†	*n* (rate per 10 000 children)†
2008–2009	19 884 (69)	7215 (25)
2010–2011	21 563 (74)	8005 (28)
2012–2013	22 259 (75)	8682 (29)
2014–2015	24 146 (80)	9749 (32)
2016–2017	24 509 (79)	10 488 (34)
Age group		
0–1 years	2845 (15)	5187 (36)
2–3 years	28 193 (166)	10 672 (62)
4–5 years	32 182 (189)	10 324 (59)
6–7 years	18 756 (110)	6722 (40)
8–9 years	11 186 (67)	4383 (30)
10–11 years	7772 (45)	3125 (23)
12–13 years	5848 (32)	2256 (15)
14–15 years	5579 (29)	1470 (10)
Gender		
Male	60 837 (89)	26 897 (41)
Female	51 524 (78)	17 242 (29)
Socioeconomic disadvantage quintiles by postcode of residence		
1 (most disadvantaged)	17 895 (65)	5440 (21)
2, 3 and 4	62 461 (88)	21 524 (33)
5 (least disadvantaged)	31 912 (87)	17 141 (49)
Remoteness by postcode of residence		
Major cities	82 742 (84)	35 364 (38)
Regional	22 974 (83)	6892 (27)
Outer regional/remote	6580 (72)	1859 (22)
Hospital type	*n* (%)	*n* (%)
Tertiary paediatric	8987 (8)	2636 (6)
Metropolitan public	14 381 (12.8)	4322 (9.8)
Regional/rural public	19 526 (17.4)	5347 (12.1)
Private	69 467 (61.8)	31 834 (72.1)
Adjuvant surgical procedures during admission		
Nose or sinus	11 734 (10.4)	11 073 (25.1)
Ear	24 931 (22.2)	26 887 (60.9)
Pre‐existing chronic conditions^	4953 (4.4)	1374 (3.1)

† Based on population data reported by the Australian Bureau of Statistics. Population rates by age, gender, socioeconomic disadvantage and remoteness are based on the latest census data (2016) resulting from dividing the number of surgeries performed in 2016 by the NSW population in each demographic strata; Children with recorded diagnosis of metabolic disorders, obesity, neoplasm of digestive or respiratory system, neuromuscular disease, chromosomal anomalies, tracheostomy or gastrostomy, coagulopathies, chronic kidney disease, craniofacial anomalies, severe developmental delay, severe cardiac or pulmonary disease. Percentages may not add to 100 due to missing values.

The most common indications for ADT (primary diagnosis) included diseases of tonsils and adenoids (68%), followed by sleep‐disordered breathing (22%) and ear conditions (5%). For AD, ear conditions were predominant (44%), followed by diseases of tonsils and adenoids (29%) and other respiratory conditions (22%; Table S1). The proportion of ADT for diseases of tonsils and adenoids decreased from 77% in 2008–2009 to 64% in 2016–2017 and those for sleep‐disordered breathing increased from 17% to 26%.

**Fig. 2:**
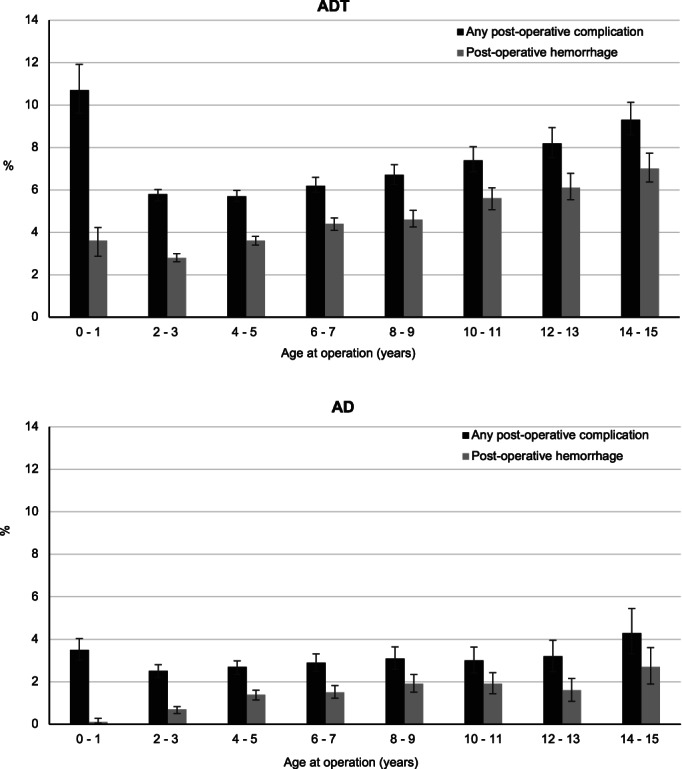
Post‐operative complications by age in children undergoing adenoid and tonsil removal in NSW, 2008–2017.

Overall, 6.5% (*n* = 7262) of children who had an ADT and 2.9% (*n* = 1276) having an AD had a post‐operative complication. Haemorrhage was the most common post‐operative complication during the initial admission for ADT and AD, and the most common in 30‐day readmissions following ADT, with respiratory complications the most common in readmissions following AD (Table 2). Compared to children undergoing ADT or AD alone, those having adjuvant procedures had higher rates of post‐operative complications (ADT: 10.5% vs. 3.4% had post‐operative haemorrhage and 12.8% vs. 5.8% had any complication; AD: 4.0% vs. 0.2% had haemorrhage and 5.5% vs. 2.0% had any complication). For ADT, standardised rates of any complications were highest in children aged 0–1 years (10.7%) and 14–15 years (9.3%), while post‐operative haemorrhage remained low for children aged 0–1 years (3.6%) and increased with age to 7.0% in children aged 14–15 years (Fig. 2). A similar pattern was observed in children having AD. Any post‐operative complications in children with adjuvant nose procedures were double of those with ADT or AD alone or with adjuvant ear procedures (ADT: 12.8% vs. 5.8%; AD: 5.5% vs. 1.8%; Fig. S1). There was also a slight increase in overall rates of post‐operative haemorrhage and any complications following ADT over time, which was consistent across all age groups (Fig. S2).

**Table 2:** Post‐operative outcomes of children undergoing tonsil and/or adenoid removal in NSW, 2008–2017

	Adenotonsillectomy, *N* = 112 361	Adenoidectomy, *N* = 44 139
Post‐operative complications	*n* (%)	*n* (%)
During initial admission		
Any haemorrhage	1351 (1.2)	470 (1.06)
Haemorrhage requiring procedure	362 (0.32)	41 (0.09)
Respiratory	128 (0.11)	18 (0.04)
Infection	11 (0.01)	–
From anaesthesia	115 (0.1)	24 (0.05)
From drug therapy	99 (0.09)	15 (0.03)
Other	76 (0.07)	7 (0.02)
Any complication	2018 (1.8)	615 (1.4)
30‐day readmissions		
Any haemorrhage	3306 (2.9)	74 (0.17)
Haemorrhage requiring procedure	687 (0.61)	23 (0.05)
Volume depletion/dehydration	503 (0.45)	25 (0.06)
Respiratory	642 (0.57)	275 (0.62)
Gastrointestinal	338 (0.30)	48 (0.11)
Infections	158 (0.14)	10 (0.02)
Symptomatic (e.g. pain and vomit)	674 (0.60)	75 (0.17)
Other	515 (0.46)	231 (0.52)
Any readmission	5452 (4.9)	695 (1.6)
Time to first readmission		
Same day of discharge	481 (8.8)	165 (23.7)
1–3 days	1215 (22.3)	96 (13.8)
4–7 days	2191 (40.2)	82 (11.8)
8–30 days	1565 (28.7)	352 (50.7)
Composite outcome†		
Any haemorrhage	4573 (4.1)	534 (1.2)
Any complication	7262 (6.5)	1276 (2.9)
Subsequent operation or reoperation		
Adenotonsillectomy	2828 (2.5)	2694 (6.1)
Adenoidectomy	1492 (1.3)	2700 (6.1)

^†^ Composite of complications during initial admission and 30‐day readmissions. Composite outcomes numbers may not equal the sum of specific post‐operative complications due to some children having multiple complications.

– denotes <5; Numbers of children having haemorrhage requiring procedure are a subset of any haemorrhage.

**Fig. 3:**
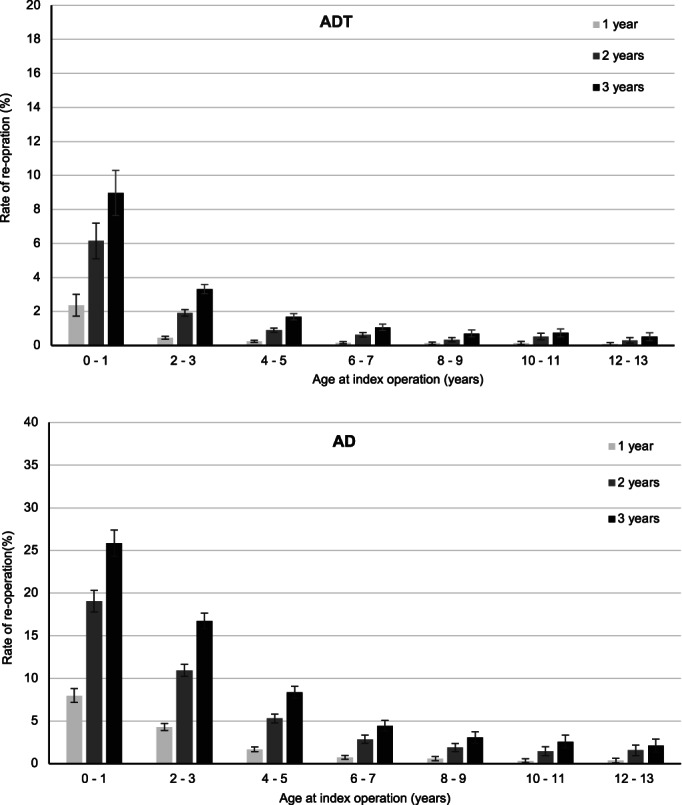
Rate of reoperation within 1, 2 and 3 years following ADT and AD by age at operation in NSW, 2008–2017. AD, adenoidectomy; ADT, adenotonsillectomy.

A total of 3.8% (*n* = 4320) children had reoperation following ADT, two‐thirds involving a repeat ADT. Of children having an AD, 12.2% (*n* = 5394) had a reoperation with half a repeat AD. Overall, 3‐year standardised reoperation rate was 1.9% (95% CI: 1.8%–2.0%) and 10.3% (95% CI: 10.0%–10.7%) following ADT and AD, respectively, and the rate of reoperation was highest among those operated at younger ages. Three‐year reoperation rate was the highest for children aged 0–1 years with 9.0% (95% CI: 7.6%–10.3%) following ADT and 25.9% (95% CI: 24.3%–27.4%) following AD, decreasing thereafter to 0.5% (95% CI: 0.3%–0.7%) and 2.1% (95% CI: 1.4%–2.9%) in children aged 12–13 years (Fig. 3). Children having an adjuvant procedure of the nose had higher 3‐year reoperation rate following ADT (4.5%; 95% CI: 0.2%–4.9%) and AD (13.5%; 95% CI: 13.0%–14.1%).

## Discussion

In 2017, 1 in every 125 Australian children had an ADT and 1 in 300 had an AD with rates increasing over the last decade. These results appear to be largely driven by an increase in surgery among younger children. The main indications for surgery were diseases of tonsils and adenoids and sleep‐disordered breathing. Of all operations, 6.5% of children undergoing ADT and 2.9% of children having an AD had a post‐operative complication with complication rates highest in youngest children aged 0–1 years and adolescents, 13–15 years, and those undergoing adjuvant procedures. About 2% of all children undergoing ADT and 10% of children having an AD required reoperation with highest rates among children having initial operation at 0–1 years.

There is wide variation in tonsillectomy rates with a 13‐fold difference reported across 31 countries, ranging from 23 to 293 per 100 000 citizens.^12^ While our rates are one of the highest and increasing over the last decade, a national study also highlighted variation by local geographical areas with an up to sixfold difference in rates of paediatric ADT in 2017–18.^11^ Our findings also highlight higher rates of ADT in children from least disadvantaged areas, consistent with national report.^11^ These results suggest differences in availability of operative services and in health‐care systems, as well as possible differences in the application of these operations by specialists in private care.

The recent rising trend in rates of ADT and AD particularly among younger aged children has also been reported in other countries including Scotland^13^ and Australia,^11^ while rates of AD have remained stable in Denmark,^14^ and ADT rates have decreased in the US in recent years.^15^ One of the main factors that has been attributed to the increase in ADT is the growing number of young children being diagnosed with, and treated for, OSA or less severe forms of disordered breathing.^16^ This may be a result of parental and clinical concerns of potential negative neurocognitive and developmental effects of sleep apnoea in young children,^3^ combined with a culture of high expectations on the benefits of surgery in the community and financial incentives for surgeons under current fee‐for‐service reimbursement schemes.^17^

The rising trend in ADT is of concern given our results of 6.5% and 2.9% of children experiencing a post‐operative complication following ADT and AD, respectively. While both our study and other similar studies^1^, ^18^ only included readmissions to hospitals, complication rates of up 20% have been reported when including non‐admitted ED presentations or any unplanned visits to health‐care providers.^19^ We also found a strong effect of age on post‐operative complications with highest rates for infants and adolescents. Studies have reported increased post‐operative haemorrhage in children aged 11–17 years^20^ and greater infections and respiratory complications for infants aged 0–1 years. We also found at least double the overall complication rates in children undergoing additional surgical procedures of the nose, due to increased haemorrhage rates. This suggests a significant influence of these procedures on the post‐operative complication rates of ADT and AD, which likely can be attributed to haemorrhage from the nose procedure site. For example, a child undergoing AD and nose procedure is almost three times more likely to have post‐operative haemorrhage than a child undergoing AD alone. These findings are important for informed decision‐making by parents and clinicians regarding the need for surgery, especially as ADT is the most common operation in children,^1^ has one of the highest rates of post‐operative complications^1^ and is the most common reason for children presenting to the emergency department for uncontrolled post‐operative pain.^21^

Our study found that 2% and 10% of children required reoperation following ADT and AD, respectively. Previous population‐based studies reported similar reoperation rates between 1.3% and 2.5% where these combined index ADT with AD.^10^, ^22^ Reoperation rates were particularly higher among children having initial surgery at younger ages, with up to one in five children under 4 years having AD requiring reoperation within 3 years. Post‐operative clinical assessment also suggests that tonsil and adenoid regrowth after surgical removal is higher among younger children.^23^ Thus, more judicious decision‐making regarding operations is required at younger ages. Consideration of long‐term outcomes is also required with adverse health outcomes, including increased risk of respiratory, infectious and allergic diseases in adulthood identified.^9^

The effectiveness of ADT or AD in children on some patient's relevant outcomes also remains questionable. For children aged 3–9 years without major medical comorbidities and with mild to moderate OSA, clinical trials have reported that, compared with no surgery, those undergoing ADT had significant reduction in Apnoea/Hypopnoea Index and Oxygen Desaturation Index scores using polysomnography, an increase in quality of life scores, sleep quality and behaviour between 7‐month and 1‐year post‐surgery. But, no benefit was found in objective measures of short‐term attention and neurocognitive outcomes compared with watchful waiting.^7^, ^8^ A systematic review also found modest and short‐term reduction in throat infections after ADT^5^. For children undergoing AD, studies suggest small beneficial effects on resolution of otitis media with effusion and decreased hearing loss.^24^

Given our findings that children younger than 2 years are at increased risk from post‐operative complications and future reoperations, alternative management strategies should be considered for them. Where possible, delaying surgery could allow time for the pathology causing symptoms to resolve naturally, assisted by non‐operative management. Some non‐operative medical therapies have shown promising effectiveness for treating OSA or adenoid hypertrophy in children and may be considered as an alternative to surgery, for young children with mild symptoms.^25^ In particular, intranasal leukotriene receptor antagonist Montelukast has shown to have moderate short‐term reduction in the number of sleep apnoeas for non‐obese otherwise healthy children with OSA.^26^ Understanding and increasing clinician and parental awareness of the risk of post‐operative complication and future reoperation and potential adverse effects are important for informed decision‐making.

For children with recurrent tonsillitis, guidelines recommend tonsil removal following the Scottish Intercollegiate Guideline Network (SIGN) criteria.^27^ However, adherence to these criteria has been poor with a study reporting that only 12% of children aged less than 15 years undergoing ADT in the UK met the SIGN criteria.^28^ Although we did not have information on tonsillitis episodes presenting to primary and specialist care to assess the appropriateness of ADT for chronic tonsillitis, these findings suggest that some operations for children with tonsillitis may be unnecessary and further monitoring of indication and appropriateness of operations are required.

The strengths of this study include the use of administrative population‐based linked data, ensuring complete ascertainment and follow‐up of children, and the generalizability of findings. Limitations of the study include the lack of information on primary care presentations which also allows the identification of post‐operative complications not involving hospital care. Therefore, our results most likely underestimate the total rates of post‐operative complications. There may have been some misclassification in the indications for surgery recorded in the hospital admissions data. For some children, some indications may have been incomplete, missing or may not fully represent the underlying diagnosis for surgery. These inconsistencies may be partially due to a lack of corresponding codes in the ICD‐10‐AM for indications of sleep‐disordered breathing or snoring. We were also unable to assess the appropriateness of recorded primary indications and we did not have information on the surgical technique used or the surgeons' experience to assess their impact on outcomes.

## Conclusion

Population rates of tonsil and adenoid removal have increased in the last decade, highest in children aged 3–4 years. Rates of ADT were also higher among children from least disadvantaged areas, residing in metropolitan areas and undergoing operations in private hospitals. Post‐operative complications and reoperation were highest in children younger than 2 years. These findings highlight that ADT and AD are not without risk especially for children at young ages. Measures to prevent unnecessary surgery are needed, including improvements in identifying children who are most likely to benefit from surgery, and prioritising non‐operative approaches and alternatives as the first line of management to ensure informed decision‐making for surgery and optimal health outcomes for children.

## Supporting information


**Figure S1** Post‐operative complications by presence of adjuvant procedures in children undergoing adenoid and tonsil removal in NSW, 2008–2017

**Figure S2** Trend in rates of post‐operative complications in children undergoing adenoid and tonsil removal in NSW, 2008–2017

**Table S1** Indications for surgery in children undergoing tonsil and adenoid removal in NSW, 2008–2017
