# Supplementary material for: Adenotonsillectomy and adenoidectomy in children: The impact of timing of surgery and post‐operative outcomes
Source: J Paediatr Child Health. 2022 Jun 3;58(9):1608–15. doi: 10.1111/jpc.16052 (PMC9543311; doi:10.1111/jpc.16052)
Supplement: Supplementary file 2 — Figure S2 Trend in rates of post‐operative complications in children undergoing adenoid and tonsil removal in NSW, 2008–2017 [file JPC-58-1608-s003.docx]

**Supplementary figure 2**: Trend in rates of post-operative complications in children undergoing adenoid and tonsil removal in NSW, 2008-2017

Rates based on composite of post-operative complications during the initial admission and 30-day readmissions
