# Supplementary material for: Adenotonsillectomy and adenoidectomy in children: The impact of timing of surgery and post‐operative outcomes
Source: J Paediatr Child Health. 2022 Jun 3;58(9):1608–15. doi: 10.1111/jpc.16052 (PMC9543311; doi:10.1111/jpc.16052)
Supplement: Supplementary file 3 — Table S1 Indications for surgery in children undergoing tonsil and adenoid removal in NSW, 2008–2017 [file JPC-58-1608-s001.pdf]

Supplementary Table 1: Indications for surgery in children undergoing tonsil and adenoid removal in NSW, 2008-2017

	**ADT N=112,361**		**AD N=44,139**	
	**Primary diagnosis**	**Any diagnosis field**	**Primary diagnosis**	**Any diagnosis field**
	**N (%)**	**N (%)**	**N (%)**	**N (%)**
**Diseases of tonsils and adenoids**	76761 (68.3)		12929 (29.4)	
Chronic tonsillitis	48672 (43.3)	56007 (49.9)	387 (0.9)	747 (1.7)
Hypertrophy of tonsils and adenoids	21918 (19.5)	37546 (33.4)	1001 (2.3)	2043 (4.6)
Hypertrophy of adenoids	4233 (3.8)	9400 (8.4)	29 (0.1)	110 (0.3)
Hypertrophy of tonsils	1040 (0.9)	9797 (8.7)	11489 (26)	25071 (56.8)
Acute tonsillitis	898 (0.8)	1239 (1.1)	23 (0.1)	63 (0.1)
**Sleep Disordered breathing**	25017 (22.3)		1690 (3.8)	
Obstructive sleep apnoea syndrome	20345 (18.1)	31705 (28.2)	1088 (2.5)	1738 (3.9)
Sleep apnoea	2439 (2.2)	4071 (3.6)	138 (0.3)	282 (0.6)
Mouth breathing or other breathing problems	1937 (1.7)	7433 (6.6)	457 (1)	2012 (4.6)
Other sleep disorders	296 (0.3)	1196 (1.1)	7 (0)	64 (0.1)
**Ear conditions**	5682 (5.1)		19492 (44.2)	
Otitis media	5126 (4.6)	19128 (17)	17820 (40.4)	23855 (54.1)
Other ear conditions	556 (0.5)	4091 (3.6)	1672 (3.8)	3978 (9.0)
**Other respiratory conditions**	4462 (4.0)		9537 (21.6)	
Conditions of nose and sinuses	3060 (2.7)	9950 (8.9)	9275 (21.0)	15447 (35.0)
Other acute upper respiratory	1402 (1.2)	2989 (2.7)	262 (0.6)	619 (1.4)
**Peritonsillar abscess**	71 (0.1)	138 (0.1)	-	-
**Other diagnoses**	368 (0.3)		490 (1.1)	

– denotes <5
